# Efficacy and safety of intranasal insulin on postoperative cognitive dysfunction in elderly patients after laparoscopic radical resection of colorectal cancer: a double-blind pilot study

**DOI:** 10.3389/fnagi.2024.1375841

**Published:** 2024-06-10

**Authors:** Hailong Zhang, Liqin Zhao, Min Li, Xu Li, Ruofan Li, Di Wu

**Affiliations:** ^1^Department of Anesthesiology, Beijing Luhe Hospital, Capital Medical University, Beijing, China; ^2^Department of Anesthesiology, Beijing Ditan Hospital, Capital Medical University, Beijing, China; ^3^Department of Gastrointestinal Surgery, Beijing Luhe Hospital, Capital Medical University, Beijing, China

**Keywords:** postoperative cognitive dysfuction by postoperative complications, intranasal insulin by intranasal drug, elderly, a double-blind pilot study by pilot study, colorectal cancer

## Abstract

**Objective:**

To evaluate the efficacy and safety of intranasal insulin on postoperative cognitive dysfunction (POCD) in elderly patients after laparoscopic radical resection of colorectal cancer.

**Methods:**

Older patients scheduled for laparoscopic radical resection of colorectal cancer at Beijing Luhe Hospital, Capital Medical University, between August 2023 and November 2023, were enrolled in this double-blind pilot study. Patients were randomized to the control and insulin groups at a 1:1 ratio. The primary outcome was the rate of POCD at postoperative 7 days.

**Results:**

A total of 61 patients (30 in the insulin group) were analyzed. The insulin group had a significantly lower POCD rate compared with the control group at postoperative day 7 [4(13.3%) vs. 12 (38.7%), *p* = 0.024]. The serum levels of IL-6, TNF-α and S100β at T_2-5_ in the insulin group were significantly lower than those of the control group (IL-6: mean difference at T_2_, −4.14, *p* = 0.036; T_3_, −3.84, *p* = 0.039; T_4_, −3.37, *p* = 0.013; T_5_, −2.57, *p* = 0.042; TNF-α: mean difference at T_2_, −3.19, *p* = 0.002; T_3_, −2.35, *p* = 0.028; T_4_, −2.30, *p* = 0.019; T_5_, −1.96, *p* = 0.0181; S100β: mean difference at T_2_, −8.30, *p* = 0.019; T_3_, −23.95, *p* = 0.020; T_4_, −20.01, *p* = 0.023; T_5_, −17.67, *p* = 0.010). No insulin allergic reactions, nasal irritation, or hypoglycemic reactions were observed in either of the groups.

**Conclusion:**

Intranasal insulin may decrease the risk of POCD and inhibit the elevated serum IL-6, TNF-α, and S100β levels in elderly patients after laparoscopic radical resection of colorectal cancer, which proves that intranasal insulin may be a promising therapeutic option for POCD.

**Clinical trial registration:**

Identifier, ChiCTR2300074423.

## Background

Postoperative cognitive dysfunction (POCD) refers to a series of changes in personality, social, and cognitive abilities that occur extensively in elderly patients after surgery. The condition is characterized by issues with memory, abstract thinking, and disorientation, accompanied by social dysfunction (Evered and Silbert, 2018). General anesthesia is considered a key risk factor for POCD (Evered et al., 2018). Pathogenesis of POCD mainly involves central inflammatory response, cerebral perfusion, blood–brain barrier damage, and circadian rhythm disorders (Kruthiventi et al., 2020; Tasbihgou and Absalom, 2021). The systemic inflammatory response caused by surgery increases the plasma levels of IL-1, IL-6, TNF-α, and other inflammatory factors, which induces the inflammatory response of CNS through various mechanisms, impairs cognitive function, and causes POCD (Li et al., 2022). However, there is still no specific and effective treatment for POCD.

Insulin can improve cognitive function by reducing intracellular amyloid plaque, promoting tau hypophosphorylation, stabilizing microtubules and promoting tubulin polymerization (Zilliox et al., 2016). Insulin directly affects inflammatory cells in the brain, reduces the release of pro-inflammatory cytokine TNFα, and may have an important role in the mechanism of action of insulin-based therapies currently being considered for CNS disorders (Brabazon et al., 2018). Exogenous insulin relies primarily on intranasal administration into the brain, which has the advantages of non-invasiveness, ease of handling, rapid absorption and onset of action, and avoidance of hepatic first-pass elimination (Born et al., 2002). Preclinical and clinical evidence suggested that intranasal insulin improves memory function (Badenes et al., 2021) and can also improve functional performance in patients with Alzheimer’s disease (AD) and Parkinson’s disease (PD) (Claxton et al., 2015; Novak et al., 2019). However, there is limited clinical evidence of intranasal insulin preventing POCD in elderly patients after laparoscopic radical resection of colorectal cancer.

Therefore, this study aimed to evaluate the efficacy and safety of intranasal insulin on POCD in elderly patients after laparoscopic radical resection of colorectal cancer.

## Methods

### Study design and participants

This double-blind pilot study enrolled elderly patients scheduled for laparoscopic radical resection of colorectal cancer at Beijing Luhe Hospital, Capital Medical University, between August 2023 and November 2023. Inclusion criteria were: (1) age ≥ 65 years old; (2) body mass index (BMI) of 18–25 kg/m^2^; (3) American Society of Anaesthesiologists (ASA) grade of I to III; (4) undergoing elective laparoscopic radical resection of colorectal cancer under general anesthesia. Exclusion criteria: (1) history of diabetes mellitus, neurological disorders, psychiatric disorders; (2) insulin allergy; (3) difficulties in communication; (4) preoperative Mini-Mental State Examination (MMSE) scores <17 for illiterate (uneducated) patients, < 20 for patients with elementary education (≤6 years of education), and < 24 for patients with secondary education or higher (>6 years of education) who were uncooperative with the investigator (Chua et al., 2019); (5) severe postoperative complications affect neurocognitive function testing.

This study was approved by the Ethics Committee of Beijing Luhe Hospital, Capital Medical University, and all patients signed written informed consent.

### Randomization and blinding

A random number table was generated using SPSS Statistics version 23.0. The patients were randomly divided into the control group and the insulin group at a ratio of 1:1. Each vial of insulin or placebo was labeled with a sequentially allocated randomization number. Participants and researchers were both blinded to the treatment allocation.

### Intervention

Patients in the insulin group were administrated 20 U of rapid-acting insulin (Gansulin, Tonghua Dongbao Pharmaceutical Co., Ltd., China) twice a day via nasal atomizers (MAD Nasal TM, Teleflex Incorporated, United States) in each nostril starting 2 days before surgery at 7:00 am and 7:00 pm; the medicine was given one more time at 1 h before admission to the operating room. Patients in the control group were administrated 0.5 mL of normal saline intranasally at the corresponding time points.

The anesthetic management was consistent for both cohorts of patients. Non-invasive blood pressure (NIBP), oxygen saturation (SpO_2_), electrocardiograph (ECG), and end-tidal carbon dioxide (PETCO_2_) were monitored after admission to the operating room. Right radial artery puncture and catheterization were conducted under local anesthesia, and right internal jugular vein puncture and catheterization were performed under ultrasound guidance. The patients received total intravenous anesthesia during surgery. After surgery, patient-controlled analgesia were used. The regimen of anesthesia and analgesia is decided by the anaesthesiologists based on patients’condition. At the end of the operation, after the patient had clear consciousness, spontaneous respiratory recovery, inspiratory SpO_2_ > 95%, which lasted for more than 5 min, swallowing reflex and conjunctival reflex being active, the endotracheal tube was removed.

### Endpoints

The primary endpoint was the rate of POCD at postoperative 7 days. Cognitive function tests were performed using neuropsychological testing methods acknowledged by the International Study Group of Postoperative Cognitive Dysfunction (ISPOCD) at preoperative enrollment (T_0_) and postoperative day 7 (T_6_). If two or more tests suggested functional deficits, the patient was considered to be with POCD (Zhang et al., 2018). Secondary endpoints included the serum interleukin-6 (IL-6), tumor necrosis factor-α (TNF-α), and S100β at admission (T_1_), end of surgery (T_2_), postoperative day 1 (T_3_), postoperative day 3 (T_4_), postoperative day 5 (T_5_). At T_1_, T_2,_ T_3_, T_4,_ and T_5_, 5-ml blood samples were drawn from the internal jugular vein. The samples were centrifuged at 3,500 rpm for 10 min, and the upper serum was collected to detect the serum IL-6, TNF-α, and S100β by ELISA. All serum biological markers were measured by enzyme-linked immunosorbent assay kit (R&D systems, Bio-Techne Ltd., UK) according to the manufacturer’s instructions. For this study, the intra- and inter-assay CVs were 4.1 and% 6.9% for IL-6, 3.7% and 6.2% for TNF-α, 4.5% and 7.1% for S100β. Safety evaluations included insulin allergic reactions, nasal irritation, and hypoglycemic reactions after drip.

### Sample size calculation

According to previous reports (Zhao et al., 2020), the incidence of POCD was 40% in the first postoperative week. Our pilot trial determined that the incidence of POCD decreased to approximately 10% when insulin was used, with α = 0.05 and β = 0.20; the required sample size was estimated to be 25 in each group. Considering a loss-to-follow-up rate of approximately 20%, 32 participants were enrolled in each group.

### Statistical analysis

The statistical analysis was performed using SPSS Statistics version 23.0 (IBM, Armonk, NY, United States). The primary analysis was prespecified to be performed in the per-protocol population. Continuous data with a normal distribution were described as means ± standard deviations (SDs) and analyzed using Student’s t-test; otherwise, they were presented as medians (interquartile range, IQR) and analyzed using the Wilcoxon rank-sum test. Categorical data were described as *n* (%) and analyzed using the chi-square test or Fisher’s exact test. A two-sided *p*-value <0.05 was considered statistically significant.

## Results

**Figure 1:**
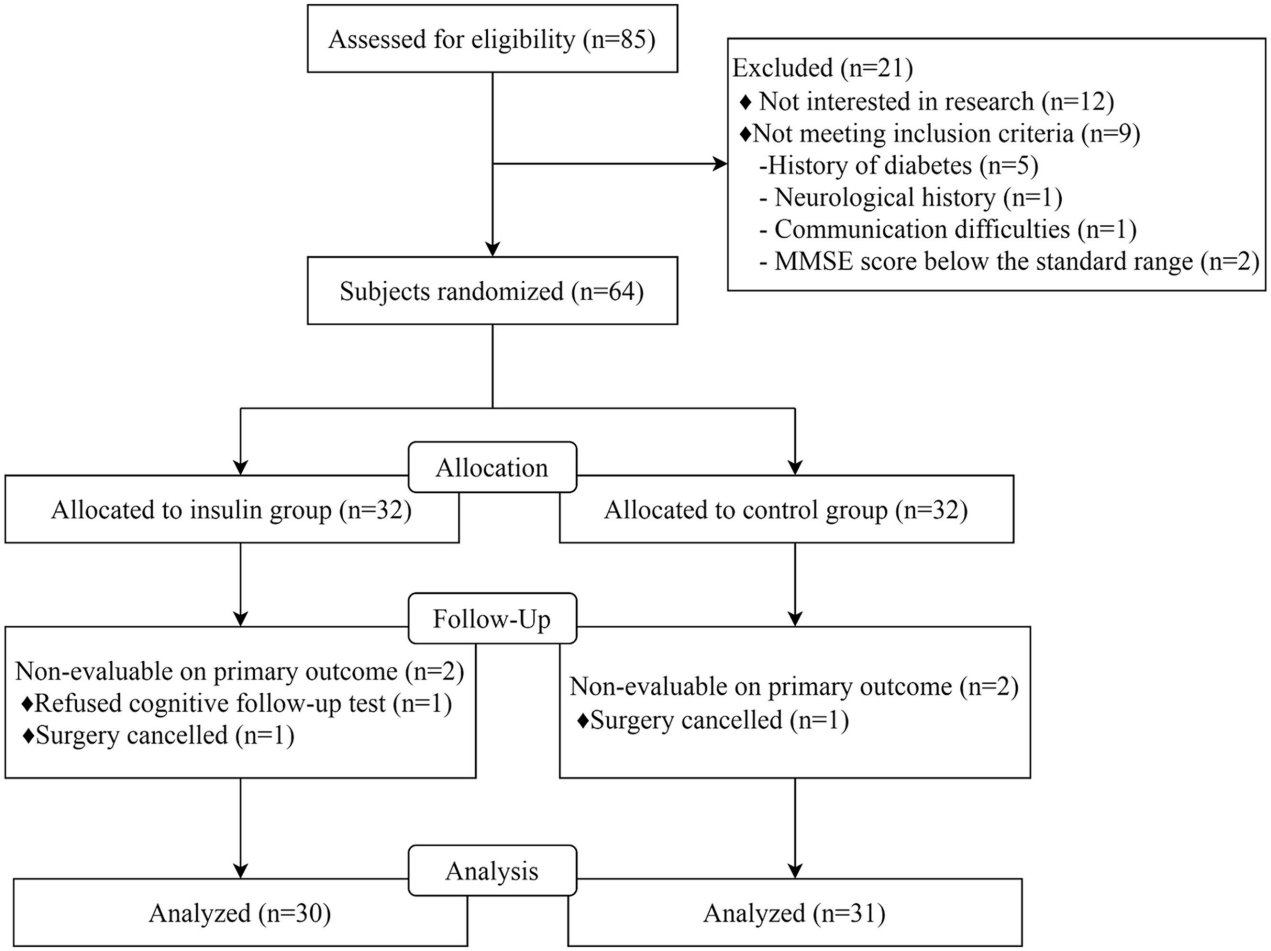
Study flowchart.

Among 85 patients screened between August 2023 and November 2023, 12 were not interested in this research, 5 had diabetes mellitus, 1 had neurological disease, 1 had communication difficulty, and 2 had MMSE scores below the standard range. A total of 64 patients were included in the final analysis, with 32 patients being allocated to the insulin group and 32 to the control group. One patient was excluded due to rejection to participate in cognitive follow-up, and 1 was excluded due to surgery being canceled in the insulin group. One patient was also excluded from the control group due to surgery being canceled, resulting in 61 patients (including 30 in the insulin group) being enrolled in this study (Figure 1). The baseline characteristics between the insulin and control groups were comparable, including age, gender ratio, body mass index (BMI), ASA grade, TNM stage, MMSE scores, education years, and operation time between the control group and insulin group (all *p* > 0.05, Table 1).

**Table 1:** Baseline characteristics.

Metrics	Insulin group (*n* = 32)	Control group (*n* = 32)	*t*	effect size	*p*- value
Male, *n* (%)	20 (63)	22 (69)			0.580
Age (years), mean ± SD	70.7 ± 4.2	69.9 ± 3.9	0.738	0.197	0.464
Body mass index (kg/m^2^), mean ± SD	22.1 ± 3.5	21.6 ± 3.2	0.175	0.149	0.679
ASA grade, *n* (%)					0.796
I	6 (19)	7 (22)			
II	22 (69)	20 (62)			
III	4 (12)	5 (16)			
TNM stage, *n* (%)					0.668
II	21 (66)	19 (59)			
III	11 (34)	13 (41)			
MMSE scores	27.9 ± 3.1	28.4 ± 2.9	0.190	−0.167	0.564
Education (years), mean ± SD	6.7 ± 3.2	6.6 ± 2.9	−0.148	0.033	0.883
Operation time (min), mean ± SD	216.6 ± 68.1	213.5 ± 64.7	0.287	0.047	0.851

The insulin group had a significantly lower POCD rate compared with the control group at postoperative day 7 [4 (13.3%) vs. 12 (38.7%), *p* = 0.024] (Table 2). There was no significant difference in all neuropsychological test parameters between the insulin and control groups at T_0_. The mental control (mean difference, 5.03, 95% CI 0.73–9.33, *p* = 0.023) and digit symbol (mean difference, 4.67, 95% CI 1.06–8.28, *p* = 0.012) in the insulin group was significantly higher than that of the control group at T_6_, and the Trail A (mean difference, −16.27, 95% CI -30.13--2.40, *p* = 0.022) was significantly lower than that of the control group at T_6_ (Supplementary Table S1).

**Table 2:** Outcome of POCD.

	Insulin group (*n* = 30)	Control group (*n* = 31)	*p*-value
1	26	19	
2	3	8	
3	1	3	
4	0	1	
≥5	0	0	
POCD patients (with 2 or more deficits)	4 (13.3%)	12 (38.7%)	0.024

**Figure 2:**
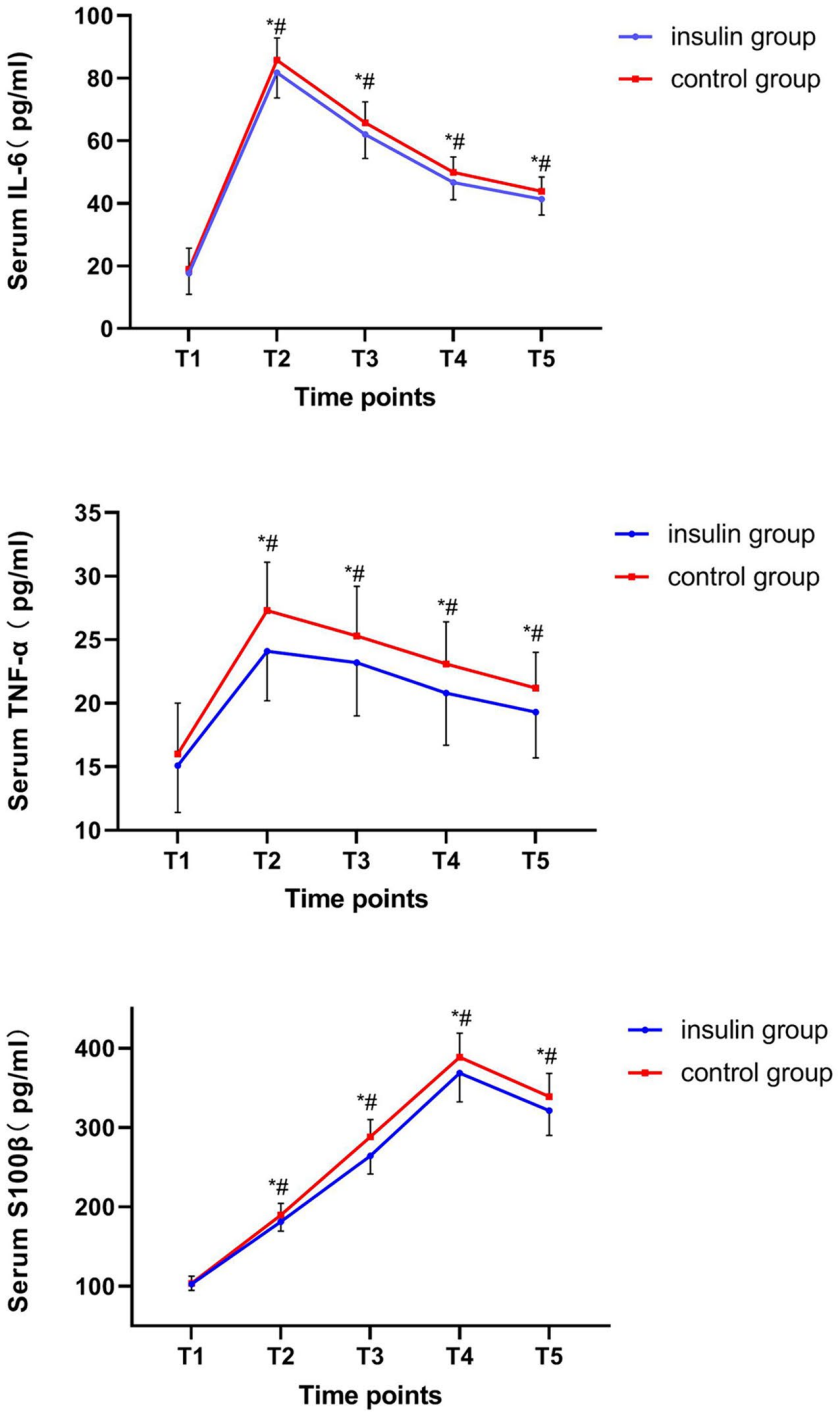
Comparison of serum interleukin (IL)-6, tumor necrosis factor (TNF)-α, and S100β protein at each time point. ^#^*p* < 0.05 vs. T_1_. *p < 0.05 vs. the control group.

The serum IL-6 and TNF-α increased from T_1_ to T_2_ and decreased from T_2_ to T_5._ Moreover, the S100β levels increased from T_1_ to T_4_ and decreased from T_4_ to T_5._ Besides, the serum levels of IL-6, TNF-α and S100β at T_2-5_ in the insulin group were significantly lower than those of the control group (IL-6: mean difference at T_2_, −4.14, *p* = 0.036; T_3_, −3.84, *p* = 0.039; T_4_, −3.37, *p* = 0.013; T_5_, −2.57, *p* = 0.042; TNF-α: mean difference at T_2_, −3.19, *p* = 0.002; T_3_, −2.35, *p* = 0.028; T_4_, −2.30, *p* = 0.019; T_5_, −1.96, *p* = 0.0181; S100β: mean difference at T_2_, −8.30, *p* = 0.019; T_3_, −23.95, *p* = 0.020; T_4_, −20.01, *p* = 0.023; T_5_, −17.67, *p* = 0.010; Figure 2). No insulin allergic reactions, nasal irritation, or hypoglycemic reactions were observed in either of the groups. The perioperative complications, such as pneumonia, incision infection, postoperative bleeding, cricoarytenoid joint dislocation, post-nausea and vomiting (PONV), fever, chills, acute renal insufficiency, and surgery-related complications, did not differ between the two groups.

## Discussion

This study found that intranasal insulin significantly reduced the risk of POCD and decreased the serum IL-6, TNF-α and S100β in older patients compared to patients treated with intranasal normal saline after laparoscopic radical resection of colorectal cancer, which provides a basis and support for preoperative intranasal insulin in older patients to improve cognitive function and decrease the risk of POCD.

Insulin is a small protein secreted by pancreatic β-cells and composed of 51 amino acid residues, which is known to regulate neural development and neuronal activities and has a key role in learning and memory (Chen et al., 2017). Currently, intranasal insulin has been reported as a treatment and prevention of postoperative delirium (POD) with a similar pathogenesis to AD (Craft et al., 2020; Nitchingham et al., 2021). It has been pointed out that POD and POCD sometimes occur in the same individual with overlapping risk factors, which suggests a common underlying neuropathogenesis (Daiello et al., 2019). Chen et al. (2017) showed that daily intranasal insulin for 3 days prior to anesthesia completely prevented the spatial learning and memory deficits in 3xTg-AD mice, which may restore insulin signaling, including GSK-3β disturbed by anesthesia via activating PI3K/PDK1/AKT pathway, and attenuate hyperphosphorylation of tau at multiple AD-associated sites (Kawano et al., 2016; Zhang et al., 2016; Li et al., 2019). Moreover, intranasal insulin can bypass the blood–brain barrier and enter the brain via olfactory and trigeminal-associated extracellular pathways and perivascular pathways, thus avoiding the side effects of peripheral insulin administration and contributing to memory improvement (Dhuria et al., 2010). Benedict et al. (2007) showed that intranasal insulin improved recall ability and self-confidence in cognitive tasks after 8-week treatment with intranasal insulin in healthy individuals. Kellar et al. (2022) found that patients in the insulin group had increased interferon-γ and eotaxin, and reduced IL-6 in cerebrospinal fluid (CSF) over the 12-month trial compared to the placebo group, suggesting alleviated neuroinflammation in cognitive impairment. Craft et al. found that insulin treatment improved delayed memory and preserved caregiver-rated functional ability, associated with changes in the Aβ42 level and the tau protein-to-Aβ42 ratio in CSF (Craft et al., 2012).

Khan et al. found that IL-6, TNF-α, and S100β were associated with poor cognitive function (Khan et al., 2020). It was also found that IL-6, TNF-α, and S100β levels were up-regulated after open cardiac surgery (Tang et al., 2017), which indicated neurological damage (Goettel et al., 2017; Liu et al., 2018). Prior literatures have demonstrated the relationship between POCD related to S100β and insulin resistance (D’Cunha et al., 2019; He et al., 2019). Huang et al. (2021) found that repeated preoperative intranasal insulin could reduce serum levels of TNF-a, IL-1, and IL-6 and prevented the development of POD after laparoscopic radical gastrointestinal surgery in elderly patients; the incidence rates of adverse events were comparable between the two groups. Huang et al. (2023) also found that preoperative intranasal insulin could significantly reduce the risk of POD in older patients undergoing radical esophagectomy, similar to this study’s results.

The present study still has several limitations. First, this study did not collect patients’ blood glucose and serum kalium information during the perioperative period to ensure safety. Secondly, this was a single-center study, and the sample size was relatively small, which limited the extrapolation of the conclusion to the general population. Third, patients often developed POCD from a few days to several months, while this study only focused on changes at postoperative 7 days and did not follow up long-term outcomes, thus failing to provide enough evidence to support the long-term efficacy of intranasal insulin on POCD. Fourthly, the biomarkers were collected from serum, which may not be the best choice for detecting biomarkers compared to CSF. Fifthly, the effects of dose and frequency of insulin administration on POCD were not investigated. Finally, this study did not explore the relationship between types of anesthetics and POCD. Although this study is a pilot study, which was conducted at a single institution on a homogeneous group of patients, our postoperative findings suggest a potentially positive role for intranasal insulin to prevent postoperative cognitive impairment and confirm that further study is warranted.

## Conclusion

Intranasal insulin may decrease the risk of POCD and inhibit the elevated serum IL-6, TNF-α, and S100β levels in elderly patients after laparoscopic radical resection of colorectal cancer, which proves that intranasal insulin may be a promising therapeutic option for POCD. A large-sample multicenter randomized trial is needed to investigate the dose and frequency of insulin administration in POCD.

## Data availability statement

The original contributions presented in the study are included in the article/Supplementary material, further inquiries can be directed to the corresponding author.

## Ethics statement

The studies involving humans were approved by Beijing Luhe Hospital, Capital Medical University (2023-LHKY-060-02). The studies were conducted in accordance with the local legislation and institutional requirements. The participants provided their written informed consent to participate in this study.

## Author contributions

HZ: Data curation, Writing – original draft, Writing – review & editing. LZ: Data curation, Writing – original draft, Writing – review & editing. ML: Formal analysis, Writing – original draft, Writing – review & editing. XL: Formal analysis, Writing – original draft, Writing – review & editing. RL: Data curation, Writing – original draft, Writing – review & editing. DW: Data curation, Writing – original draft, Writing – review & editing.

## References

[ref1] Badenes R. Qeva E. Giordano G. Romero-García N. Bilotta F. (2021). Intranasal insulin administration to prevent delayed neurocognitive recovery and postoperative neurocognitive disorder: a narrative review. Int. J. Environ. Res. Public Health 18:2681. doi: 10.3390/ijerph18052681, PMID: 33799976 PMC7967645

[ref2] Benedict C. Hallschmid M. Schultes B. Born J. Kern W. (2007). Intranasal insulin to improve memory function in humans. Neuroendocrinology 86, 136–142. doi: 10.1159/000106378 17643054

[ref3] Born J. Lange T. Kern W. McGregor G. P. Bickel U. Fehm H. L. (2002). Sniffing neuropeptides: a transnasal approach to the human brain. Nat. Neurosci. 5, 514–516. doi: 10.1038/nn849, PMID: 11992114

[ref4] Brabazon F. Bermudez S. Shaughness M. Khayrullina G. Byrnes K. R. (2018). The effects of insulin on the inflammatory activity of BV2 microglia. PLoS One 13:e0201878. doi: 10.1371/journal.pone.0201878, PMID: 30148836 PMC6110462

[ref5] Chen Y. Dai C. L. Wu Z. Iqbal K. Liu F. Zhang B.. (2017). Intranasal insulin prevents anesthesia-induced cognitive impairment and chronic neurobehavioral changes. Front. Aging Neurosci. 9:136. doi: 10.3389/fnagi.2017.00136, PMID: 28539885 PMC5424543

[ref6] Chua X. Y. Choo R. W. M. Ha N. H. L. Cheong C. Y. Wee S. L. Yap P. L. K. (2019). Mapping modified Mini-mental state examination (MMSE) scores to dementia stages in a multi-ethnic Asian population. Int. Psychogeriatr. 31, 147–151. doi: 10.1017/s1041610218000704 30017004

[ref7] Claxton A. Baker L. D. Hanson A. Trittschuh E. H. Cholerton B. Morgan A.. (2015). Long acting intranasal insulin Detemir improves cognition for adults with mild cognitive impairment or early-stage Alzheimer's disease dementia. J. Alzheimers Dis. 45, 1269–1270. doi: 10.3233/jad-159002 25869922

[ref8] Craft S. Baker L. D. Montine T. J. Minoshima S. Watson G. S. Claxton A.. (2012). Intranasal insulin therapy for Alzheimer disease and amnestic mild cognitive impairment: a pilot clinical trial. Arch. Neurol. 69, 29–38. doi: 10.1001/archneurol.2011.233, PMID: 21911655 PMC3260944

[ref9] Craft S. Raman R. Chow T. W. Rafii M. S. Sun C. K. Rissman R. A.. (2020). Safety, efficacy, and feasibility of intranasal insulin for the treatment of mild cognitive impairment and Alzheimer disease dementia: a randomized clinical trial. JAMA Neurol. 77, 1099–1109. doi: 10.1001/jamaneurol.2020.1840, PMID: 32568367 PMC7309571

[ref10] Daiello L. A. Racine A. M. Yun Gou R. Marcantonio E. R. Xie Z. Kunze L. J.. (2019). Postoperative delirium and postoperative cognitive dysfunction: overlap and divergence. Anesthesiology 131, 477–491. doi: 10.1097/aln.0000000000002729 31166241 PMC6692220

[ref11] D’Cunha N. M. McKune A. J. Panagiotakos D. B. Georgousopoulou E. N. Thomas J. Mellor D. D.. (2019). Evaluation of dietary and lifestyle changes as modifiers of S100β levels in Alzheimer's disease. Nutr. Neurosci. 22, 1–18. doi: 10.1080/1028415x.2017.1349032, PMID: 28696163

[ref12] Dhuria S. V. Hanson L. R. Frey W. H. (2010). Intranasal delivery to the central nervous system: mechanisms and experimental considerations. J. Pharm. Sci. 99, 1654–1673. doi: 10.1002/jps.21924 19877171

[ref13] Evered L. A. Silbert B. S. (2018). Postoperative cognitive dysfunction and noncardiac surgery. Anesth. Analg. 127, 496–505. doi: 10.1213/ane.0000000000003514 29889707

[ref14] Evered L. Silbert B. Knopman D. S. Scott D. A. DeKosky S. T. Rasmussen L. S.. (2018). Recommendations for the nomenclature of cognitive change associated with Anaesthesia and Surgery-20181. J. Alzheimers Dis. 66, 1–10. doi: 10.3233/jad-189004, PMID: 30347621

[ref15] Goettel N. Burkhart C. S. Rossi A. Cabella B. C. Berres M. Monsch A. U.. (2017). Associations between impaired cerebral blood flow autoregulation, cerebral oxygenation, and biomarkers of brain injury and postoperative cognitive dysfunction in elderly patients after major noncardiac surgery. Anesth. Analg. 124, 934–942. doi: 10.1213/ane.0000000000001803, PMID: 28151820

[ref16] He X. Long G. Quan C. Zhang B. Chen J. Ouyang W. (2019). Insulin resistance predicts postoperative cognitive dysfunction in elderly gastrointestinal patients. Front. Aging Neurosci. 11:197. doi: 10.3389/fnagi.2019.00197, PMID: 31440156 PMC6694405

[ref17] Huang Q. Li Q. Qin F. Yuan L. Lu Z. Nie H.. (2021). Repeated preoperative intranasal Administration of Insulin Decreases the incidence of postoperative delirium in elderly patients undergoing laparoscopic radical gastrointestinal surgery: a randomized, placebo-controlled, double-blinded clinical study. Am. J. Geriatr. Psychiatry 29, 1202–1211. doi: 10.1016/j.jagp.2021.02.043 33757723

[ref18] Huang Q. Shi Q. Yi X. Zeng J. Dai X. Lin L.. (2023). Effect of repeated intranasal Administration of Different Doses of insulin on postoperative delirium, serum τ and Aβ protein in elderly patients undergoing radical esophageal Cancer surgery. Neuropsychiatr. Dis. Treat. 19, 1017–1026. doi: 10.2147/ndt.S405426, PMID: 37144143 PMC10153451

[ref19] Kawano T. Iwata H. Aoyama B. Nishigaki A. Yamanaka D. Tateiwa H.. (2016). The role of hippocampal insulin signaling on postoperative cognitive dysfunction in an aged rat model of abdominal surgery. Life Sci. 162, 87–94. doi: 10.1016/j.lfs.2016.08.020, PMID: 27561842

[ref20] Kellar D. Register T. Lockhart S. N. Aisen P. Raman R. Rissman R. A.. (2022). Intranasal insulin modulates cerebrospinal fluid markers of neuroinflammation in mild cognitive impairment and Alzheimer's disease: a randomized trial. Sci. Rep. 12:1346. doi: 10.1038/s41598-022-05165-3, PMID: 35079029 PMC8789895

[ref21] Khan B. A. Perkins A. J. Prasad N. K. Shekhar A. Campbell N. L. Gao S.. (2020). Biomarkers of delirium duration and delirium severity in the ICU. Crit. Care Med. 48, 353–361. doi: 10.1097/ccm.0000000000004139, PMID: 31770149 PMC7242000

[ref22] Kruthiventi S. C. Laporta M. L. Deljou A. Knopman D. S. Petersen R. C. Schroeder D. R.. (2020). Preoperative cognitive impairment associated with oversedation during recovery from anesthesia. J. Anesth. 34, 390–396. doi: 10.1007/s00540-020-02764-0 32222908

[ref23] Li X. Run X. Wei Z. Zeng K. Liang Z. Huang F.. (2019). Intranasal insulin prevents anesthesia-induced cognitive impairments in aged mice. Curr. Alzheimer Res. 16, 8–18. doi: 10.2174/1567205015666181031145045, PMID: 30381076

[ref24] Li Z. Zhu Y. Kang Y. Qin S. Chai J. (2022). Neuroinflammation as the underlying mechanism of postoperative cognitive dysfunction and therapeutic strategies. Front. Cell. Neurosci. 16:843069. doi: 10.3389/fncel.2022.843069, PMID: 35418837 PMC8995749

[ref25] Liu X. Yu Y. Zhu S. (2018). Inflammatory markers in postoperative delirium (POD) and cognitive dysfunction (POCD): a meta-analysis of observational studies. PLoS One 13:e0195659. doi: 10.1371/journal.pone.0195659, PMID: 29641605 PMC5895053

[ref26] Nitchingham A. Milne A. Toson B. Tuch B. Agar M. Close J.. (2021). Intranasal insulin for treatment of delirium in older hospitalised patients: study protocol for a randomised controlled trial. BMJ Open 11:e050765. doi: 10.1136/bmjopen-2021-050765, PMID: 34667006 PMC8527126

[ref27] Novak P. Pimentel Maldonado D. A. Novak V. (2019). Safety and preliminary efficacy of intranasal insulin for cognitive impairment in Parkinson disease and multiple system atrophy: a double-blinded placebo-controlled pilot study. PLoS One 14:e0214364. doi: 10.1371/journal.pone.0214364, PMID: 31022213 PMC6483338

[ref28] Tang N. Jiang R. Wang X. Wen J. Liu L. Wu J.. (2017). Insulin resistance plays a potential role in postoperative cognitive dysfunction in patients following cardiac valve surgery. Brain Res. 1657, 377–382. doi: 10.1016/j.brainres.2016.12.027, PMID: 28048971

[ref29] Tasbihgou S. R. Absalom A. R. (2021). Postoperative neurocognitive disorders. Korean J. Anesthesiol. 74, 15–22. doi: 10.4097/kja.20294 32623846 PMC7862941

[ref30] Zhang Y. Dai C. L. Chen Y. Iqbal K. Liu F. Gong C. X. (2016). Intranasal insulin prevents anesthesia-induced spatial learning and memory deficit in mice. Sci. Rep. 6:21186. doi: 10.1038/srep21186, PMID: 26879001 PMC4754754

[ref31] Zhang Y. Shan G. J. Zhang Y. X. Cao S. J. Zhu S. N. Li H. J.. (2018). Propofol compared with sevoflurane general anaesthesia is associated with decreased delayed neurocognitive recovery in older adults. Br. J. Anaesth. 121, 595–604. doi: 10.1016/j.bja.2018.05.059, PMID: 30115258

[ref32] Zhao W. Hu Y. Chen H. Wang X. Wang L. Wang Y.. (2020). The effect and optimal dosage of Dexmedetomidine plus Sufentanil for postoperative analgesia in elderly patients with postoperative delirium and early postoperative cognitive dysfunction: a single-center, prospective, randomized, double-blind, controlled trial. Front. Neurosci. 14:549516. doi: 10.3389/fnins.2020.549516, PMID: 33192244 PMC7645155

[ref33] Zilliox L. A. Chadrasekaran K. Kwan J. Y. Russell J. W. (2016). Diabetes and cognitive impairment. Curr. Diab. Rep. 16:87. doi: 10.1007/s11892-016-0775-x 27491830 PMC5528145

