# Supplementary material for: Efficacy and safety of intranasal insulin on postoperative cognitive dysfunction in elderly patients after laparoscopic radical resection of colorectal cancer: a double-blind pilot study
Source: Front Aging Neurosci. 2024 Jun 10;16:1375841. doi: 10.3389/fnagi.2024.1375841 (PMC11194343; doi:10.3389/fnagi.2024.1375841)
Supplement: Supplementary file 1 [file Table_1.docx]

**Supplementary table 1.** Comparison of neuropsychological assessment scores

| Metrics | Group | T_0_ | Difference (95  % CI) | t | effect size | *P*  *value* | T_6_ | Difference (95  % CI) | t | effect size | *P*  *value* |
| --- | --- | --- | --- | --- | --- | --- | --- | --- | --- | --- | --- |
| Mental control | Insulin group  (n=30) | 82.4 ± 9.4 | 0.20 (-3.73, 4.12) | 0.707 | 0.183 | 0.482 | 78.8 ± 9.6 | 5.03 (0.73, 9.33) | 2.338 | 0.606 | 0.023 |
|  | Control group(n=31) | 80.7 ± 9.2 |  |  |  |  | 73.7 ± 7.1 |  |  |  |  |
| Visional rational | Insulin group  (n=30) | 9.2 ± 2.3 | 0.17 (-0.99, 1.33) | 0.292 | 0.044 | 0.772 | 8.3 ± 2.4 | 0.59 (-0.60, 1.78) | 0.994 | 0.261 | 0.324 |
|  | Control group(n=31) | 9.1 ± 2.2 |  |  |  |  | 7.7 ± 2.2 |  |  |  |  |
| Paired associate verbal learning | Insulin group  (n=30) | 18.0 ± 2.6 | 0.74 (-0.60, 2.08) | 1.110 | 0.275 | 0.272 | 15.1 ± 2.8 | 1.23 (-0.18, 2.64) | 1.751 | 0.436 | 0.085 |
|  | Control group(n=31) | 17.3 ± 2.5 |  |  |  |  | 13.9 ± 2.7 |  |  |  |  |
| Digit span forward | Insulin group  (n=30) | 8.1 ± 1.6 | -0.03 (-0.88, 0.82) | -0.071 | 0.000 | 0.943 | 7.0 ± 1.5 | 0.32 (-0.45, 1.09) | 0.841 | 0.200 | 0.404 |
|  | Control group(n=31) | 8.1 ± 1.7 |  |  |  |  | 6.7 ± 1.5 |  |  |  |  |
| Digit span backward | Insulin group  (n=30) | 4.2 ± 1.0 | -0.07 (-0.46, 0.60) | 0.271 | 0.000 | 0.787 | 3.9 ± 1.0 | 0.45 (-0.03, 0.92) | 1.895 | 0.421 | 0.063 |
|  | Control group(n=31) | 4.2 ± 1.1 |  |  |  |  | 3.5 ± 0.9 |  |  |  |  |
| Digit symbol | Insulin group  (n=30) | 28.1 ± 7.8 | 0.20 (-3.73, 4.12) | 0.100 | 0.026 | 0.920 | 24.7 ± 8.0 | 4.67 (1.06, 8.28) | 2.587 | 0.666 | 0.012 |
|  | Control group(n=31) | 27.9 ± 7.4 |  |  |  |  | 20.0 ± 6.0 |  |  |  |  |
| Trails A | Insulin group  (n=30) | 108.0 ± 25.6 | 3.48 (-9.02, 15.98) | 0.558 | 0.130 | 0.579 | 115.8 ± 22.3 | -16.27 (-30.13, -2.40) | -2.347 | -0.610 | 0.022 |
|  | Control group(n=31) | 104.5 ± 28.2 |  |  |  |  | 132.0 ± 30.1 |  |  |  |  |
| Pegboard favored hand | Insulin group  (n=30) | 82.9 ± 9.5 | 2.80 (-2.16, 7.76) | 1.130 | 0.288 | 0.263 | 86.3 ± 9.3 | -2.80 (-6.91, 1.32) | -1.361 | -0.348 | 0.179 |
|  | Control group(n=31) | 80.1 ± 9.9 |  |  |  |  | 89.1 ± 6.6 |  |  |  |  |
| Pegboard unfavored hand | Insulin group  (n=30) | 81.1 ± 10.3 | -1.06 (-6.44, 4.32) | -0.395 | -0.105 | 0.694 | 83.6 ± 11.3 | -4.14 (-9.36, 1.08) | -1.587 | -0.412 | 0.118 |
|  | Control group(n=31) | 82.2 ± 10.7 |  |  |  |  | 87.8 ± 9.0 |  |  |  |  |

Neurocognitive function tests specifically included: mental control, visual regeneration, paired visual retention learning, and digit span-forward/backward in the Wechsler Adult Memory Scale (revised), digit symbol in the Wechsler Adult Intelligence Scale (revised), with higher scores on the above test items representing better function; trail retention test A and grooved pegboard- favored/unfavored hand, with lower scores on these two tests representing better function. First, the quantitative learning effect was obtained by subtracting the basal test value of the control normal subjects from the test value after 7 days; then the preoperative basal test value of the patients was subtracted from the test value at 7 days after surgery, and then the average learning effect was subtracted, divided by the standard deviation of the learning effect of the control group to obtain the Z value of each test, and the Z score was higher than 1.96 (two standard deviations), suggesting that there was a deficit in this neurological function.
